# Soil-transmitted helminth infection in school age children in Sierra Leone after a decade of preventive chemotherapy interventions

**DOI:** 10.1186/s40249-019-0553-5

**Published:** 2019-07-02

**Authors:** Yakuba Mohamed Bah, Mohamed Salieu Bah, Jusufu Paye, Abdulai Conteh, Sam Saffa, Alie Tia, Mustapha Sonnie, Amy Veinoglou, Joseph J. Amon, Mary Hamer Hodges, Yaobi Zhang

**Affiliations:** 1 Neglected Tropical Disease Control Program, Ministry of Health and Sanitation, Freetown, Sierra Leone; 2 Helen Keller International, Freetown, Sierra Leone; 3 Helen Keller International, New York, USA; 4 Helen Keller International, Regional Office for Africa, Yoff-Dakar, Senegal

**Keywords:** Soil transmitted helminths, Sierra Leone, Mass drug administration, Epidemiological coverage, Impact assessment, Water, sanitation and hygiene

## Abstract

**Background:**

Baseline mapping of soil-transmitted helminth (STH) infections among school age children (SAC) in 2008–2009 found high or moderate prevalence in 13 of the 14 districts in Sierra Leone. Following these surveys, mass drug administration (MDA) of mebendazole/albendazole was conducted biannually at national level targeting pre-school children (PSC) aged 12–59 months and intermittently at sub-national level targeting SAC. In addition, MDA with ivermectin and albendazole for eliminating lymphatic filariasis (LF) has been conducted nationwide since 2010 targeting individuals over 5 years of age. Each MDA achieved high coverage, except in 2014 when all but one round of MDA for PSC was cancelled due to the Ebola emergency. The objective of the current study was to determine the prevalence and intensity of STH infections among SAC after a decade of these deworming campaigns.

**Methods:**

Seventy-three schools in 14 districts were purposefully selected, including 39 schools from the baseline surveys, with approximately two sites from each of low, moderate and high prevalence categories at baseline per district. Fresh stool samples were collected from 3632 children aged 9–14 years (male 51%, female 49%) and examined using the Kato Katz technique.

**Results:**

The prevalence of STH infections in Sierra Leone decreased in 2016 compared to 2008: *Ascaris lumbricoides* 4.4% (95% confidence interval [*CI*]: 3.7–5.1%) versus 6.6% (95% *CI*: 0–25%), *Trichuris trichiura* 0.7% (95% *CI*: 0.5–1.1%) versus 1.8% (95% *CI*: 0–30.2%), hookworm 14.9% (95% *CI*: 13.8–16.1) versus 38.5% (95% *CI*: 5.4–95.1%), and any STH 18.3% (95% *CI*:17.0–19.5%) versus 48.3% (*CI*: 5.4–96.3%), respectively. In 2016, no district had high hookworm prevalence and four districts had moderate prevalence, compared with eight and four districts respectively in 2008. In 2016, the arithmetic mean hookworm egg count in all children examined was light: 45.5 eggs per gram (EPG) of faeces, (95% *CI*:\ 35.96–55.07 EPG); three (0.08%) children had heavy infections and nine (0.25%) children had moderate infections.

**Conclusions:**

Sierra Leone has made considerable progress toward controlling STH as a public health problem among SAC. As LF MDA phases out (between 2017 and 2021), transition of deworming to other platforms and water and sanitation strategies need to be strengthened to maintain STH control and ultimately interrupt transmission.

**Electronic supplementary material:**

The online version of this article (10.1186/s40249-019-0553-5) contains supplementary material, which is available to authorized users.

## Multilingual abstracts

Please see Additional file 1 for translations of the abstract into the five official working languages of the United Nations.

## Background

Soil-transmitted helminths (STH) contribute a substantial health and socioeconomic burden on poor communities in sub-Saharan Africa [1]. The most common species are *Ascaris lumbricoides* (roundworm)*, Trichuris trichiura* (whipworm) and hookworm. STH are associated with stunted growth, impaired cognition, poor school performance and weak gross domestic productivity [2–5]. Heavy hookworm infections contribute to anemia and lead to increased maternal morbidity and low birth weight rates [6–9], perpetuating a vicious cycle of poor health and poverty [10].

In 2001, the World Health Assembly called on endemic countries to control STH [11] targeting pre-school children (PSC) aged 12–59 months, school age children (SAC) aged 5–14 years and at-risk adults including women of reproductive age. The World Health Organization (WHO) recommends mass drug administration (MDA) for STH twice yearly in high risk communities (prevalence over 50%) and annually in moderate risk communities (prevalence between 20 and 50%) [12, 13]. Longer-term control strategies include improved access to safe water and sanitation and behaviour changes in personal and community hygiene, such as hand washing with soap and avoidance and safe disposal of human faeces [14].

In the 1990s, individual studies in Sierra Leone reported that STH was widely endemic, with moderate to high prevalence and heavy intensity of infection with frequent polyparasitism [15–19]. *Necator americanus* was determined as the predominant species of hookworm [20] and the national prevalence was tied with Togo for the highest rate in Africa [21]. Deworming of SAC demonstrated a positive impact on growth in the local context [22–24].

During the war (1991–2002), MDA began targeting displaced persons in camps where security allowed, and immediately post-war, MDA targeted SAC in schools prioritized according to district nutritional status whilst studies continued to show mostly moderate to high levels of infection [25, 26].

In 2005, the national onchocerciasis control program started community directed treatment with ivermectin (CDTI) to everyone over five years of age (excluding pregnant women) in all meso- and hyper-endemic communities [27, 28] and antenatal clinics began routine deworming of pregnant women from their second trimester [29].

**Fig. 1:**
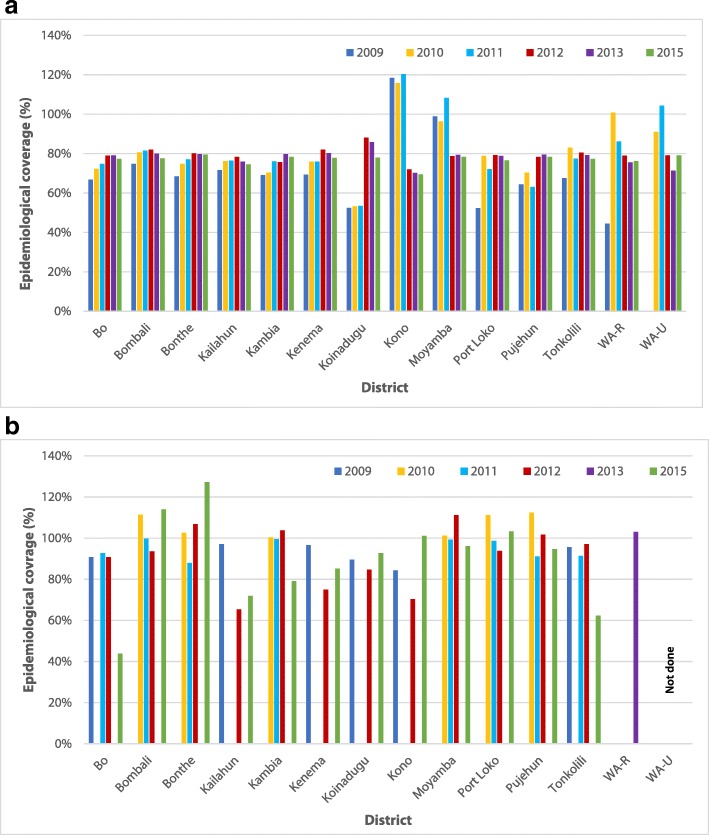
Epidemiological coverage (%) of MDA by district and by year. **a**. LF MDA targeting population of five years and over; **b**. STH MDA targeting SAC only. WA-U: Western Area Urban; WA-R: Western Area Rural; MDA: Mass drug administration

Nationwide biannual MDA for PSC commenced in 2006 with mebendazole (2006–2009), and then continued with albendazole. The program consistently reported over 85% treatment coverage which was validated by post-event coverage surveys [30]. In 2007, distribution of albendazole for LF treatment was integrated with CDTI in eight districts and by 2010 had reached all 14 districts including the Western Area Rural and Urban as shown in Fig. 1a [31].

In 2008, the national baseline mapping for STH was performed among SAC and results showed that the overall prevalence was low for *A. lumbricoides* (7.2%) and *T. trichiura* (3.9%) and moderate for hookworm (38.5%) and any STH (49.6%) [32]. In 2009, a supplementary survey in seven districts found the overall intensities of infections were light for all three species: *A. lumbricoides* (17.8 eggs per gram [EPG] of faeces), *T. trichiura* (20.3 EPG), hookworm (53.0 EPG) [33].

Since 2009, a second round of school-based MDA for SAC has been performed periodically at sub-national level either during MDA for schistosomiasis or independently, with reported coverage consistently over 75% as validated by surveys as shown in Fig. 1b [34, 35].

In total, by 2016 at least 13 rounds of MDA for PSC and five rounds of LF MDA had been completed in all 14 districts, in addition to the periodic, sub-national deworming campaigns targeting SAC. Over 48 million doses of ivermectin, mebendazole or albendazole were distributed between 2008 and 2016. An impact assessment was conducted in 2016 to assess the prevalence and intensity of STH infection among SAC after a decade of deworming. This paper presents the results and discusses the impact of mass deworming campaigns on STH prevalence and intensity compared to baseline and the transition strategies to maintain STH control as LF MDA phases out in Sierra Leone.

## Methods

### Study area and population

Sierra Leone is a small (71 740 km^2^) tropical country in West Africa bordered by Guinea, Liberia and the Atlantic with a diverse environment ranging from savanna in the north to rainforests in the east and south. The climate is characterized by a dry season in the winter months (November to April) and a rainy season in the other months. The annual rainfall averages from 2000 to 3000 mm. The geography is relatively flat in the west and hilly and mountainous in the north-east, with an altitude of just under 2000 m. In 2016 the population of Sierra Leone was estimated at 7292546 million and with 1 968 987 SAC.

About 16 ethnic groups (Muslim: 77%, Christians: 23%) live in Sierra Leone with Krio (English-based creole) being the most widely spoken language. Seventy-five percent of SAC are enrolled in primary education [36]. Over 75% of rural households rely on agriculture for their livelihood, however low productivity contributes to chronic food insecurity and malnutrition. Mining of diamonds, bauxite, gold, rutile and iron provides employment in some districts, but despite a wealth of these natural resources 96% of households are classified within the lowest- and low-income global quintiles [37]. Thirty-eight percent of households (50% in rural areas) are still reliant on an unimproved water source for drinking, of which 31% fetch water from a river or stream. In ten districts, less than 10% of households have access to improved sanitation [38].

### Baseline (2008 and 2009) surveys

Survey methods for the baseline surveys conducted in 2008 and 2009 have been described previously [39]. Briefly, in 2008, 52 schools were selected purposefully and 100 children per school (50 males, 50 females) aged 8–16 years randomly selected (*n* = 2777) with a mean age of 10.39 ± 2.32 years [24]. In 2009, 59 schools in seven districts were selected with 30 children aged 9–14 years per school were selected (*n* = 1760) with a mean age of males: 11.52 ± 1.67 and females: 11.3 ± 1.68 years [25].

### Impact survey (2016)

#### Survey sites

A school-based, cross-sectional survey was conducted 8 months after the last LF MDA in all 14 districts. The sample size was not calculated but followed the WHO recommendations for impact assessments for STH in SAC at sentinel sites (one site per 200 000–300 000 SAC) [13]. For better geographical and epidemiological representation, two sites from each of low, moderate and high prevalence categories at baseline were purposefully selected from each district. This computed to four to six sites per district and a total of 73 sites (schools) across the country, as some districts could not provide all three categories of baseline prevalence. Fifty percent of the sentinel sites were from those originally surveyed at baseline and the others were newly selected as spot check sites to avoid bias from repeatedly sampling the same sites. The geographical coordinates of each site were recorded using handheld global positioning system (GPS) devices.

#### Sampling and data collection

Fifty children (25 males and 25 females) aged 9–14 years were randomly selected from classes 4, 5 and 6. Each child was given a plastic container to provide a fresh stool sample in the morning and allocated a unique identification number. Each child’s name, sex and age was recorded. All stool samples were examined on site within 5 h of collection by the Kato-Katz method using a 41.7 mg thick faecal smear template and microscopy (one slide per sample). The number of eggs identified per slide was multiplied by 24 and recorded as EPG [40]. All positive slides and 10% of all negative slides were re-examined by a senior technician for quality assurance.

### Data analysis

Parasitological data were recorded in Microsoft excel (version 10, Microsoft, Redmond, US) and exported into SPSS (version 23, IBM, Armonk, US) for analysis. District-level and national-level estimates of intensity of infection were calculated using arithmetic mean egg counts and 95% confidence intervals (*CI*) were calculated taking into consideration the cluster nature of school children using district as strata and school as clusters. Chi-squared test was used to compare infection prevalence between males and females and baseline versus 2016, and the Kruskal Wallis test was used to compare the mean egg counts. Maps were drawn using ArcGIS software (version 10.4, ESRI, Redlands, US). Prevalence of STH were classified as low (less than 20%), moderate (between 20 and 50%) or high (50% and above). Intensity was classified in EPG by species as light, moderate or heavy for *A. lumbricoides:* 1–4999; 5000–49 999; ≥50 000; *T. trichiura,* 1–999; 1000–9999; ≥10 000 or hookworm: 1–1999; 2000–3999; and ≥ 4000 respectively.

For estimates of MDA epidemiological coverage rates, treatment data reported by the Ministry of Health and Sanitation (MoHS) was compared with the total at-risk population for each age group estimated according to the projections from the 2004 national population census and adjusted for rural-urban migration as appropriate from the annual community census.

## Results

A total of 3632 children agreed to participate in the study (mean age 11.4 ± 1.5 years, 51% male) and provided stool samples for examination from 73 sites (39 original sentinel and 44 spot check) in all 14 districts.

### Prevalence

Overall prevalence of all species and any STH was low, *A. lumbricoides:* 4.4% (95% *CI*: 3.7–5.1%)*, T. trichiura:* 0.7% (95% *CI*: 0.5–1.1%), hookworm: 14.9% (95% *CI*: 13.8–16.1%) and any STH: 18.3% (95% *CI*: 17.0–19.5%) (Table 1). Prevalence by district ranged from 0 to 8.8% for *A. lumbricoides,* from 0 to 13.9% for *T. trichiura,* from 1.2 to 33.1% for hookworm and from 6.6 to 34.7% for any STH. There were no districts with high prevalence and six districts (Bombali, Bonthe, Koinadugu, Moyamba, Pujehun and Tonkolili) with moderate prevalence.

**Table 1:** Soil-transmitted helminth prevalence (%) with 95% *CI* in school-age children in 2008 and in 2016 in Sierra Leone

	Prevalence in 2008* (95% *CI*)					Prevalence in 2016 (95% *CI*)					Reduction compared to 2008 (%)	
	*n*	*Ascaris lumbricoides*	*Trichuris trichiura*	Hookworm	Any STH	*n*	*A. lumbricoides*	*T. trichiura*	Hookworm	Any STH	Hookworm	Any STH
Overall	2777	6.6 (0–25)	1.8 (0–30.2)	38.5 (5.4–95.1)	48.3(5.4–96.3)	3632	4.4 (3.7–5.1)^a^	0.7 (0.5–1.1)^a^	14.9 (13.8–16.1) ^a^	18.3 (17.0–19.5) ^a^	48.3%	63.1%
By District												
Bo	163	2.0 (0–11.8)	1.11 (0–3.6)	12.9 (5.4–71.4)	18.4 (5.4–71.4)	294	0.0 (0.0–1.3)	0.3 (0.1–1.9)	8.2 (5.6–11.9)	8.2 (5.6–11.9)	59.4%	67.5%
Bombali	215	8.6 (6.0–12.5)	0 (0–1.2)	17.9 (13.3–29.7)	25.2(18.1–40.5)	244	3.7 (2.0–6.9)	2.9 (1.4–5.8)	25.8 (20.7–31.7)	28.3 (23.0–34.2)	−42.5%	−4.8%
Bonthe	196	1.0 (0–19.0)	13.7 (10.3–18.2)	56.5 (28.1–70.9)	62.7 (28.1–80.0)	251	2.8 (1.4–5.6)	1.2 (0.4–3.5)	33.1 (27.5–39.1)	34.7 (29.0–40.7)	41.0%	54.3%
Kailahun	187	0.0 x(0.0–0.0)	0 (0–2.2)	49.8 (32.6–52.6)	49.8 (34.8–52.6)	298	3.4 (1.8–6.1)	0.7 (0.2–2.4)	9.7 (6.9–13.6)	12.4 (9.1–16.6)	79.1%	73.7%
Kambia	223	13.6 (8.2–21.3)	0 (0–6.7)	22.5 (16.3–26.7)	35.8 24.5–48.3)	250	8.8 (5.9–13.0)	0.0 (0.0–1.5)	1.2 (0.4–3.5)	10.0 (6.9–14.3)	94.5%	73.5%
Kenema	208	0 (0–6.1)	1.8 (0–3.3)	53.3 (41.5–66.7)	55.3 (41.5–69.7)	301	0.3 (0.1–1.9)	0.3 (0.1–1.9)	6.0 (3.8–9.3)	6.6 (4.3–10.0)	88.5%	88.1%
Koinadugu	272	3.0 (0–8.6)	0 (0–3.0)	64.8 (53.7–95.1)	68.5(56.7–96.3)	300	7.7 (5.2–11.2)	0.0 (0.0–1.3)	14.0 (10.5–18.4)	20.0 (15.9–24.9)	80.9%	74.5%
Kono	244	4.8 (3.2–6.9)	0 (0–1.6)	13.7 (8.1–15.5)	18.5 (11.3–20.7)	242	5.8 (3.5–9.5)	1.7 (0.6–4.2)	12.8 (9.2–17.6)	17.4 (13.1–22.6)	−0.8%	3.3%
Moyamba	217	9.8 (6.6–12.7)	15.0 (3.4–30.2)	60.7 (42.6–70.9)	72.3 (45.9–80)	252	5.2 (3.0–8.6)	0.8 (0.2–2.8)	23.8 (19.0–29.4)	27.4 (22.2–33.2)	59.0%	66.8%
Port Loko	173	11.0 (1.7–25)	0.9 (0–3.1)	46.4 (38.5–51.7)	53.3(50–56.1)	249	8.0 (5.3–12.1)	0.0 (0.0–1.5)	4.4 (2.5–7.7)	11.2 (7.9–15.8)	90.7%	81.0%
Pujehun	198	13.4 (5.1–21.8)	3.5 (0–5.5)	42.7 (8.5–75.9)	53.6 (15.3–75.9)	252	6.3 (3.9–10.1)	0.4 (0.1–2.2)	18.7 (14.3–23.9)	21.8 (17.2–27.3)	50.0%	60.4%
Tonkolili	280	7.0 (6.7–14.6)	2.2 (1.3–10)	24.7 (17.1–35.4)	3.3 (30–50)	301	4.3 (2.5–7.2)	1.3 (0.5–3.4)	31.2 (26.3–36.7)	33.2 (28.1–38.7)	−26.8%	9.8%
WA-R	201	9.3 (4.5–13.2)	5.6 (3.6–8.4)	28.5 (25–44.7)	41.7 (31.8–60.5)	201	0.5 (0.1–2.8)	0.5 (0.1–2.8)	13.9 (9.8–19.4)	13.9 (9.8–19.4)	54.9%	70.0%
WA-U	–	–	–	–	–	197	4.6 (2.4–8.5)	0.5 (0.1–2.8)	4.6 (2.4–8.5)	9.6 (6.3–14.6)	–	–
By Sex												
Male	1411					1844	4.1 (3.3–5.1)	1.0 (0.6–1.5)	18.8 (17.1–20.7)^b^	21.5 (19.7–23.4)^b^	–	–
Female	1366					1788	4.6 (3.8–5.7)	0.5 (0.3–1.0)	10.9 (9.5–12.4)	14.9 (13.4–16.7)	–	–
By prevalence level												
Low	282	3.5 (1.9–6.4)	1.1 (0.4–3.1)	9.2 (6.4–13.2)	13.8 (10.3–18.3)	2092	2.0 (1.5–2.7)	0.3 (0.1–0.6)^c^	6.0 (5.0–7.1)^d^	8.3 (7.2–9.5)	34.8%	39.9%
Moderate	1095	7.1 (5.7–8.8)	2.6 (1.9–3.8)	23.1 (20.7–25.7)	32.9 (30.2–35.7)	1241	4.7 (3.6–6.0)	1.0 (0.6–1.8)^d^	23.4 (21.2–25.9)^d^	29.2 (26.7–31.8)	−1.3%	11.2%
High	1400	8.0 (6.7–9.5)	5.4 (4.4–6.7)	56.4 (53.7–58.9)	69.8 (67.3–72.1)	299	9.0 (6.3–12.8)	2.7 (1.4–5.2)^c^	42.1 (36.7–47.8)^c^	53.8 (48.2–59.4)	25.4%	22.9%
By Sites												
Sentinel	–	–	–	–	–	1305	3.9 (3.0–5.1)	0.5 (0.3–1.1)	13.3 (11.6–15.3)^e^	16.5 (14.6–18.6)^e^	–	–
Non- Sentinel	–	–	–	–	–	2327	4.6 (3.8–5.5)	0.9 (0.6–1.3)	15.8 (14.4–17.4)	19.3 (17.7–20.9)	–	–

* Data for prevalence of STH in 2008 are available in Koroma et al. [32] in which WA-R and WA-U were grouped into Western Area

^a^ Significantly reduced prevalence between 2008 versus 2016 (*P* < 0.001)

^b^ Significantly higher prevalence in males versus females in 2016 (*P* = 0.001)

^c^ No significant difference between baseline versus impact (*P* > 0.05)

^d^ Significantly reduced between baseline versus impact (*P* < 0.05)

^e^ No significant difference between sentinel and non-sentinel sites (*P* > 0.05)

*CI*: Confidence interval; STH: Soil-transmitted helminth; WA-U: Western Area Urban; WA-R: Western Area Rural

**Fig. 2:**
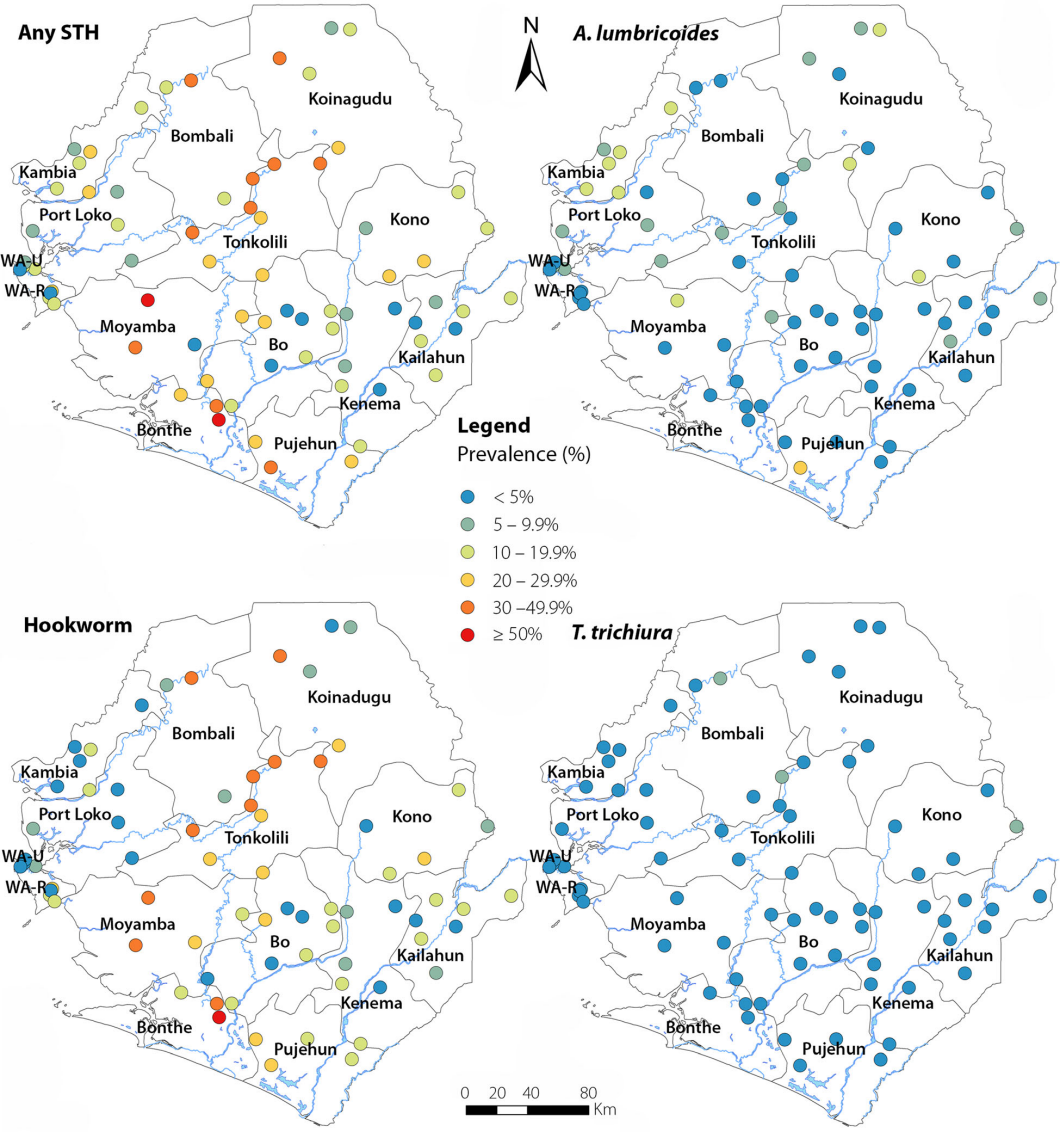
Distribution and prevalence thresholds of STH infections by survey sites in 2016 in Sierra Leone. WA-U: Western Area Urban; WA-R: Western Area Rural

The geographical location and prevalence category of each site surveyed is shown in Fig. 2. Prevalence by site ranged from 0 to 26.0% for *A. lumbricoides,* from 0 to 8.0% for *T. trichiura,* from 1.2 to 50.0% for hookworm and from 1.2 to 53.1% for any STH. Only three sites had high prevalence (in Bonthe, Moyamba and Tonkolili respectively). There was no significant difference in prevalence between sentinel sites versus spot check sites.

Compared to 2008 levels, overall prevalence of *A. lumbricoides, T. trichiura*, hookworm and any STH decreased by 38.9, 82.1, 48.3 and 63.1% respectively (*P* < 0.001 for all). Ten districts had a significant decrease in the prevalence of hookworm and 11 districts had a significant decrease in the prevalence of any STH (*P* ≤ 0.001 for both) (Table 1). Reductions in prevalence of more than 80% were observed for hookworm in four districts (Kambia, Kenema, Koinadugu and Port Loko) and for any STH in two districts (Kenema and Port Loko). Although there was a slight increase in prevalence of hookworm in Bombali and Tonkolili and in prevalence of any STH in Bombali, these increases were not statistically significant (*P* > 0.05). There was no change in either hookworm or any STH prevalence in Kono, where prevalence was low at baseline.

### Egg count

The overall arithmetic mean egg counts were light, for *A. lumbricoides:* 14.41 EPG (95% *CI* 4.60–29.43 EPG), *T. trichiura:* 3.38 EPG (95% *CI*: 0.41–10.88 EPG) and hookworm: 45.53 EPG (95% *CI*: 36.38–55.24 EPG). By district the arithmetic mean egg count ranged from 0.0 to 111.8 EPG for *A. lumbricoides,* from 0.0 to 32.1 EPG for *T. trichiura,* and from 0.5 to 163.9 EPG for hookworm (Table 2).

**Table 2:** Arithmetic mean egg count (EPG) (95% CI) in school-age children in 2016 in Sierra Leone

	Arithmetic mean EPG in 2016				*P* value 2016 vs 2009^b^		
	*n*	*Ascaris lumbricoides*	*Trichuris trichiura*	Hookworm	*A. lumbricoides*	*T. trichiura*	Hookworm
Overall	3632	14.41 (4.60–29.43)	3.38 (0.41–10.88)	45.53 (36.38–55.24)			
By District							
Bo	294	0.0 (0.0–0.0)	0.08 (0–0.24)	15.92 (6.59–25.25)	0.002	0.001	0.000
Bombali	244	58.72 (0–150.57)	3.54 (0–8.17)	163.87 (59.20–268.53)	0.000	NS	0.001
Bonthe	251	1.15 (0.16–2.14)	3.06 (0–7.77)	67.60 (43.55–91.66)			
Kailahun	298	1.61 (0.39–2.83)	32.05 (0–94.82)	18.52 (3.63–33.42)	NS	0.001	0.001
Kambia	250	7.20 (3.71–10.69)	0.0 (0.0–0.0)	0.48 (0–1.05)			
Kenema	301	2.07 (0–6.15)	0.24 (0–0.71)	3.83 (1.80–5.85)	NS	NS	0.000
Koinadugu	300	6.96 (3.62–10.30)	0.0 (0.0–0.0)	35.28 (20.03–50.53)	0.000	0.002	0.002
Kono	242	2.18 (0.83–3.54)	0.79 (0–1.71)	51.37 (0.29–102.46)	0.000	NS	0.000
Moyamba	252	6.00 (0.54–11.46)	0.19 (0–0.46)	45.81 (28.57–63.05)			
Port Loko	249	9.16 (4.32–13.99)	0.0 (0.0–0.0)	6.75 (1.63–11.87)			
Pujehun	252	23.05 (10.02–36.08)	0.19 (0–0.57)	27.43 (17.54–37.31)			
Tonkolili	301	1.59 (0.35–2.84)	2.15 (0–5.93)	152.45 (99.99–204.91)	NS	0.038	0.000
WA-R	201	0.36 (0–1.06)	0.12 (0–0.36)	25.31 (12.58–38.05)			
WA-U	197	111.84 (0–327.57)	0.24 (0–0.72)	14.01 (0–31.05)			
By sex							
Male	1844	6.33 (2.45–10.20)	6.15 (0–16.30)	62.38 (48.55–76.22)			
Female	1788	22.74 (0–49.27)	0.54 (0–1.19)	28.12 (14.99–41.25)			
Seven districts only							
Overall	1980	9.4 (0.0–20.7)	5.7 (0.0–15.2)	60.7 (43.8–77.6)	NS	0.001	0.000
Male	1009	5.8 (0.0–12.3)	10.4 (0.0–29.0)	85.9 (61.5–110.3) ^a^	NS	NS	0.000
Female	971	13.1 (0.0–35.1)	0.8 (0.0–2.0)	34.6 (11.3–57.9) ^a^	NS	0.041	NS

^a^ Significantly higher arithmetic mean epg in males versus females for hookworm in 2009 and 2016 (*P* < 0.001 for both)

^b^ Data for arithmetic mean EPG in 2009 are available in Hodges et al. [35], and not shown here

*WA-U* Western Area Urban *WA-R* Western Area Rural, *NS* Not significant

By site the arithmetic mean egg counts were light ranging from 0.0 to 432.5 EPG for *A. lumbricoides,* from 0.0 to 190.1 EPG for *T. trichiura,* and from 0.0 to 284.2 EPG for hookworm.

All individual infections for *A. lumbricoides* and *T. trichiura* were light while three children had heavy and nine had moderate hookworm infections. The combined prevalence of moderate and heavy hookworm infection was 0.3% (95% *CI*: 0.2–0.6%). There was a higher arithmetic mean egg count of hookworm infection in males (62.38 EPG, 95% *CI*: 48.55–76.22 EPG) versus females (28.12 EPG, 95% *CI*: 14.99–41.25 EPG) (*P* < 0.001).

Overall between 2009 and 2016 egg counts in the seven districts decreased for *A. lumbricoides* from 17.8 EPG (95% *CI*: 0.0–38.9) to 9.4 EPG (95% *CI*: 0.0–20.7) (*P* > 0.05) and *T. trichiura* decreased from 20.3 EPG (95% *CI*: 0.0–48.5) to 5.7 EPG (95% *CI*: 0.0–15.2) (*P* = 0.001). Hookworm increased from 53.0 EPG (95% *CI*: 38.4–67.7) but remained very light at 60.7 EPG (95% *CI*: 43.8–77.6) (*P* < 0.001) (Table 2).

## Discussion

The recent national Neglected Tropical Disease (NTD) Master Plans (2010–2015 and 2016–2020) [38, 41] aimed to control STH in SAC by the year 2020. By 2016 the overall prevalence of any STH was less than 20% and there was less than 1% moderate or heavy infections. However, six districts remained above 20% prevalence and there were no significant reductions in three districts (Bombali, Kono and Tonkolili).

Possible explanations for why some districts continue to have moderate STH prevalence may include: (1) high baseline prevalence greater than 75% (Bonthe, Moyamba and Koinadugu); (2) underestimation of the district population (Bombali, Kono, Pujehun and Tonkolili) which may have led to an underestimation of drug supplies and/or an overestimation of treatment coverage; (3) fewer rounds of SAC deworming (Bombali, Bonthe, Moyamba and Pujehun) due to funding limitations; (4) employment-seeking migration (Bombali, Kono and Moyamba) resulting in hard-to-reach communities and/or recent settlements not being recognized; (5) high proportion of households lacking access to safe drinking water (Bonthe, Kambia Moyamba and Tonkolili); and (6) high proportion of households still practicing open defecation (Bonthe, Kailahun and Pujehun) [38].

MDA coverage among PSC has ranged from 88 to 104% since 2008, which may reflect an over-estimations of previous coverage by as much as 10% [32, 42]. The national population for 2015 was projected to be 6.5 million people using an annual growth rate of 2.4% with adjustments for urbanization [43]. However, the annual growth rate was found to be 3.2% in the 2015 national census [44], with significant deviation from projections by district due to unforeseen migration, most noticeably in Bombali, Moyamba and Kono, where employment seekers have migrated for mining opportunities.

The decline in STH prevalence and mean egg counts seems to have started post-war, prior to the baseline surveys and could be attributed to the introduction of biannual deworming of PSC and CDTI in 2006, since ivermectin also affects STHs although it is more effective against *A. lumbricoides* and *T. trichiura* than hookworms [45–47].

Despite these achievements, annual MDA for STH targeting PSC and SAC should continue in the six districts with prevalence over 20% based on the most recent WHO recommendations [12, 13]. Deworming for non-pregnant adolescent girls (10–19 years of age) and non-pregnant women of reproductive age (15–49 years of age) is also recommended. Continued deworming of SAC in the other eight districts could be considered based upon the high anaemia prevalence [48] and poor water, sanitation and hygiene (WASH) conditions.

In 2017, eight districts passed the LF transmission assessment survey [49] and LF MDA was transitioned to MDA for onchocerciasis with ivermectin only. The remaining LF-endemic districts are anticipated to transition by 2020. Albendazole for SAC can be integrated with the MDA for onchocerciasis until onchocerciasis transmission is interrupted in 12 districts, which is anticipated in 2023.

From 2018, the semi-annual MDAs for PSC began transitioning to routine deworming performed by health staff based in peripheral health units and outreach stations. Maintaining effective coverage in vulnerable locations, including both remote rural areas and large urban slums, will be challenging for health staff in underserved areas. Supportive supervision and targeted coverage surveys have been planned to help ensure effective MDA coverage is reached.

As LF MDA phases out, alternative strategies for adolescent girls and non-pregnant women of reproductive age will be required [50]. However, alternative school- and/or community-based MDA platforms for SAC may also be needed to maintain control [51] in the Western Area rural and urban where there is no onchocerciasis [30]. Antenatal deworming coverage should be increased (target coverage of 80%) as a priority for both reproductive health and anaemia prevention [52, 53]. In addition, access to safe drinking water, improved latrines and increased behaviour change for personal and environmental hygiene are essential.

The Government of Sierra Leone has adopted new targets toward achieving the sustainable development goals of improving water quality and halving the proportion of untreated wastewater by 2030 [54]. Social and behaviour change communications around hand washing with soap at critical times (before eating, preparing food and after using the toilet or coming into contact with human/animal faeces) have been promoted and were intensified during the Ebola emergency in 2014–2015, but much more needs to be done with support from key opinion leaders and stakeholders within schools and communities to be effective.

Control of STH in SAC has also been reported from Burkina Faso (prevalence 1.3%), Uganda (prevalence 8.8%), and Benin (prevalence 13.8%) [55–57]*.* In Uganda, a national deworming program started in 2003 involving similar MDA strategies as in Sierra Leone and found STH control in SAC had been achieved by 2016. Cameroon also has an integrated NTD control strategy and documented an impressive decrease of 79.0% in mean prevalence of STH to 6.3% over the last decade [58]. By comparison, in Rwanda deworming of PSC started in 2004 and of SAC in 2007; however, STH transmission remained moderate (38%) in rural areas in 2014 [59].

There were certain limitations in this survey. Firstly, only one Kato-Katz slide was used for diagnosis, and the low sensitivity of this method may have underestimated the prevalence and slightly overestimated the intensity of infection. Secondly, there was a lack of egg count data from the baseline survey in 2008. Comparison of egg count data from seven districts only did not represent the overall situation in the country. Thirdly, the sample sizes for 2008, 2009 and 2016 varied: 100, 30 and 50 children per site respectively, with the mean age of participants slightly higher at baseline. This may have caused an inaccuracy in the comparison. However, considering the national strategy is STH control, such a slight misestimation would not seriously change the overall findings of this study. Lastly, the surveys were conducted in SAC only as has been customary for baseline mapping and impact assessments. The situation in other at-risk groups as defined by WHO (PSC, adolescent girls and women of reproductive age) was not studied. Further surveys to include these at-risk groups may be needed for the national NTD program to make informed decisions on the STH treatment strategy in the future.

## Conclusions

Sierra Leone has made considerable progress towards controlling STH as a public health problem in SAC at national, district and school-levels through various MDA campaigns. As LF MDA phases out, the transition to other treatment platforms needs to be considered and WASH and behaviour change communication strategies need to be strengthened to maintain the gains toward nationwide STH control and ultimately interrupt transmission.

## Additional file

Additional file 1: Multilingual abstracts in the five official working languages of the United Nations. (PDF 475 kb)

## Data Availability

All data generated or analysed during this study are included in this published article. The dataset analysed is available from the corresponding author on reasonable request and can be made available with permission from the MoHS Sierra Leone.

## Abbreviations

CDTI: Community-directed treatment with ivermectin
*CI*: Confidence interval
DHMT: District Health Management Team
LF: Lymphatic filariasis
MDA: Mass drug administration
MOHS: Ministry of Health and Sanitation
NTD: Neglected tropical diseases
NTDP: Neglected Tropical Disease Program
PHU: Peripheral health unit
PSC: Pre-school-aged children
SAC: School-aged children
STH: Soil-transmitted helminth
USAID: United States Agency for International Development
WASH: Water, sanitation and hygiene
WHO: World Health Organization
