# Supplementary material for: Soil-transmitted helminth infection in school age children in Sierra Leone after a decade of preventive chemotherapy interventions
Source: Infect Dis Poverty. 2019 Jul 2;8:41. doi: 10.1186/s40249-019-0553-5 (PMC6604471; doi:10.1186/s40249-019-0553-5)
Supplement: Supplementary file 1 — Multilingual abstracts in the five official working languages of the United Nations. (PDF 475 kb) [file 40249_2019_553_MOESM1_ESM.pdf]

Translation of the abstract into the five official working languages of the United Nations

عدوى الديدان الطفيلية المنقولة عن طريق التربة لدى الأطفال في سن المدرسة في دولة سيراليون بعد عقد من التدخلات الوقائية بالعلاج الكيماوي

يعقوب محمد باه و محمد ساليو باه و يوسف بايي و عبد الله كونته و سام سافا و علي تيا و مصطفى سوني و آمي فينوغلو و جوزيف ج. أمون و ماري همر هودجز و ياو بي زهانج.

الخلاصة:

الخلفية: أوجدت الدراسة الاستقصائية لعدوى الديدان الطفيلية المنقولة عن طريق التربة لدى الأطفال في سن المدرسة بين 2008-2009 انتشاراً متوسطاً الى عالٍ في 13 من أصل 14 مقاطعة في سيراليون، و أعطيت بعد هذه الدراسات جرعات دوائية من (المبندازول/البندازول) مستهدفةً الأطفال دون سن المدرسة (12-59 شهراً) مرتين في السنة على المستوى الوطني و بشكل متقطع للأطفال دون سن المدرسة وعلى المستوي دون الوطني. و إضافة إلى ذلك فقد أعطيت جرعات دوائية جماعية من (ايفرمكتين و البندازول) لمكافحة عدوى الديدان من نوع الفيلاريات المفاوية (lymphatic filariasis) في كامل البلاد منذ 2010 مستهدفةً الأفراد فوق سن الخامسة، و قد حققت كل حملة دوائية تغطية عالية باستثناء في عام 2014 حيث ألغيت كافة الجولات الدوائية عدا واحدة بسبب حالة الطوارئ نتيجةً لفيروس ايبولا. تهدف الدراسة الحالية إلى تحديد انتشار و كثافة عدوى الديدان الطفيلية المنقولة عن طريق التربة بين الأطفال في سن المدرسة بعد عقد من حملات مكافحة الديدان.

الطرق: اختيرت سبع و ثلاثون مدرسة في 14 مقاطعة ، من ضمنها 39 مدرسة اختيرت من الدراسة الاستقصائية مع موقعين تقريباً لكل من فئات الانتشار المنخفضة و المعتدلة و العالية و لكل مقاطعة، جمعت عينات براز حديثة من 3632 طفلاً أعمارهم ما بين التاسعة و الرابعة عشر (51% ذكور و 49% إناث) اختبرت بتقنية (الكاتو كاتز).

النتائج: تناقص في انتشار عدوى الديدان الطفيلية المنقولة عن طريق التربة في سيراليون في عام 2016 مقارنةً بعام 2008: ديدان الصفر الخراطيني 4.4% (*نطاق الثقة (CI)* 95%: 3,7-5,1%) مقابل 6,6% (*CI* 95%:0-25%)، للدودة السوطية 0,7% (*CI* 95%: 0,5-1,1%) مقابل 1,8% (*CI* 95%: 0-30,2%)، دودة الأنكلستوما 14,9 % (*CI* 95%: 13,8-16,1%) مقابل 38,5% (*CI* 95%: 5,4-95,1%) و أي ديدان منقولة عن طريق التربة 18,3% (*CI* 95%: 17,0-19,5%)، مقابل 48,3% (*CI* 95%: 5,4-96,3%) على التوالي. في عام 2016 لم يكن هناك انتشار عالٍ لدودة الأنكلستوما في أي مقاطعة بينما كان الانتشار متوسطاً في أربع مقاطعات مقارنةً بعام 2008 حيث كان الانتشار عالٍ في ثمان مقاطعات و متوسط في اربعة. و كان المتوسط الحسابي لتعداد بيوض دودة الأنكلستوما في عام 2016 لدى الأطفال المختَبَرين صغيراً: 45,5 بيضة للغرام الواحد من العينة (epg) (*CI* 95%: 35,96-55,07 epg) و كان لدى ثلاث أطفال (0,08%) عدوى ثقيلة و لدى تسع أطفال (0,25%) عدوى متوسطة.

الاستنتاجات: أحرزت سيراليون تقدماً معتبراً نحو ضبط الديدان الطفيلية المنقولة بالتربة كمشكلة صحية عامة بين الأطفال في سن المدرسة، حيث ستلغى جرعات الدواء الجماعية للفيلاريات اللمفاوية تدريجياً (بين 2017 و 2021), و ينبغي توجيهة جهود مكافحة الديدان إلى المنصات الأخرى و دعم استراتيجيات للماء و الصرف الصحي من اجل ضبط الديدان الطفيلية المنقولة بالتربة و ايقاف العدوى نهائياً.

Translated from English version into Arabic by Mustafa Keshkeia, Revised by Jaafar Jumaah, through

TRANSLATORS
WITHOUT BORDERS

塞拉利昂学龄儿童的土源性蠕虫感染情况：十年的预防性化疗干预成果评估

Yakuba Mohamed Bah, Mohamed Salieu Bah, Jusufu Paye, Abdulai Conteh, Sam Saffa, Alie Tia,

Mustapha Sonnie, Amy Veinoglou, Joseph J. Amon[3], Mary Hamer Hodges and Yao-Bi Zhang

摘要
引言：土源性蠕虫病在塞拉利昂 14 个卫生区中的 13 个都呈中度到高度的流行。随着 2008 到 2009 的调查结果，卫生部在全国范围展开对 12-59 月大的学龄前儿童进行甲苯咪唑或阿苯哒唑每年两次的大规模治疗，同时也根据药物的供应情况对学龄儿童提供治疗。另外，国家消除丝虫病项目在全国开展每年一次对 5 岁以上人群的伊维菌素和阿苯哒唑联合治疗。除了 2014 年由于 Ebola 的影响而取消大部分治疗计划外，每次治疗都取得了很高的覆盖率。本研究对大规模治疗十年来土源性蠕虫的感染情况进行了评估。
方法：在全国 14 个卫生区目标性地选择了 73 所小学，大约在每个区的低度、中度和高度流行区各选两所小学，其中 39 所小学来自治疗前开展基线调查的学校。用 Kato Katz 法对 3632 名 9 到 14 岁儿童提供的新鲜粪便标本进行了检测，其中 51%为男性、49%为女性。
结果：2016 年各种土源性蠕虫感染率与 2008 年数据相比均有所降低，其中， 蛔虫感染率从 6.6% (95% CI: 0-25%)降为 4.4% (95% CI: 3.7-5.1%)，鞭虫感染率从 1.8% (95% CI: 0-30.2%)降为 0.7% (95% CI: 0.5-1.1%)，钩虫感染率从 38.5% (95% CI: 5.4-95.1%)降为 14.9% (95% CI: 13.8-16.1)，而总感染率从 48.3% (5.4-96.3%)降为 18.3% (95% CI:17.0-19.5%)。 2016 年 14 个卫生区中没有一个呈高度钩虫感染，有 4 个区呈中度钩虫感染，而 2008 年分别为 8 个和 4 个。在所有检测的儿童中钩虫虫卵计数算术均数为 45.5 epg (95% CI: 35.96-55.07 epg)。有 3 个儿童（0.08%）呈重度感染，9 个（0.25%）呈中度感染。
结论：塞拉利昂在控制学龄儿童土源性蠕虫病方面取得了显著进步。随着消除丝虫病大规模治疗的终止（预计 2017 到 2021），为了维护蠕虫病控制成果并进一步阻断其传播，驱蠕虫治疗需要与其它平台相结合，同时提供净水和卫生策略需要加强。

Translated from English version into Chinese by Yao-Bi Zhang

Géohelminthiases parmi les enfants d'âge scolaire au Sierra Leone, après une décennie de chiomiothérapies préventives

Yakuba Mohamed Bah, Mohamed Salieu Bah, Jusufu Paye, Abdulai Conteh, Sam Saffa, Alie Tia, Mustapha Sonnie, Amy Veinoglou, Joseph J. Amon[3], Mary Hamer Hodges et Yao-Bi Zhang

Résumé
Contexte : Une cartographie initiale des géohelminthiases réalisée en 2008-2009 parmi les enfants d'âge scolaire a révélé une prévalence élevée à modérée des infestations dans 13 des 14 districts du Sierra Leone. À la suite de ces relevés, une administration en masse de mébendazole/albendazole a été réalisée en ciblant les enfants d'âge préscolaire âgés de 12 à 59 mois deux fois par an au niveau national et, par intermittences, les enfants d'âge scolaire au niveau infranational. Les habitants âgés de plus de 5 ans reçoivent en outre, depuis 2010, une administration en masse d'ivermectine et d'albendazole à l'échelle nationale, dans le but d'éliminer la filariose lymphatique. La couverture de chaque administration en masse de médicaments (AMM) a été excellente, sauf en 2014 où toutes les administrations aux enfants d'âge préscolaire, sauf une, ont été annulées à cause de l'urgence de l'épidémie d'Ébola. La présente étude avait pour but de déterminer la prévalence et l'intensité des géohelminthiases parmi les enfants d'âge scolaire après une décennie de campagnes de

vermifugation.
Méthodes : Soixante-treize écoles de 14 districts ont été choisies, dont 39 écoles incluses dans les relevés de ligne de base, en incluant deux sites de faible prévalence, deux de prévalence modérée et deux de prévalence élevée lors du relevé initial par district. Des échantillons de selles ont été recueillis extemporanément auprès de 3632 enfants âgés de 9 à 14 ans (51 % de garçons, 49 % de filles) et examinés par la technique de Kato-Katz.
Résultats : La prévalence des géohelminthiases au Sierra Leone était plus faible en 2016 qu'en 2008 : *Ascaris lumbricoides* 4,4 % (intervalle de confiance [*IC*] à 95 % de 3,7 à 5,1 %) contre 6,6 % (*IC* à 95 % de 0 à 25 %), *Trichuris trichiura* 0,7 % (*IC* à 95 %de 0,5 à 1,1 %) contre 1,8 % (*IC* à 95 % de 0 à 30,2 %), ankylostomes 14,9 % (*IC* à 95 %de 13,8 à 16,1 %) contre 38,5 % (*IC* à 95 % de 5,4 à 95,1 %) et tous géohelminthes 18,3 % (*IC* à 95 % de 17,0 à 19,5 %) contre 48,3 % (*IC* à 95 % de 5,4 à 96,3 %). En 2016, aucun district ne présentait une prévalence élevée des ankylostomiases et quatre districts présentaient une prévalence modérée, contre huit et quatre respectivement en 2008. En 2016, la moyenne arithmétique du nombre d'œufs d'ankylostomes parmi tous les enfants examinés était basse : 45,5 œufs/g (*IC* à 95 % de 35,96 à 55,7 œufs/g) ; trois enfants (0,08 %) étaient fortement infestés et neuf (0,25 %) modérément infestés.
Conclusions : Le Sierra Leone a considérablement progressé dans la lutte contre les géohelminthiases en tant que problème de santé publique parmi les enfants d'âge scolaire. Avec l'arrêt des AMM contre la filariose lymphatique (entre 2017 et 2021), il sera nécessaire de transférer la vermifugation vers d'autres plateformes et de renforcer les stratégies concernant l'eau et l'assainissement afin de continuer à juguler les géohelminthiases et, à terme, d'interrompre leur transmission.

Translated from English version into French by Suzanne Assenat, Revised by Melinda Legendre, through

TRANSLATORS
WITHOUT BORDERS

Распрострвнение почвенной гельминтной инфекции у детей школьного возраста в Сьерра-Леоне после десятилетия профилактических химиотерапевтических вмешательств

Якуба Мохамед Ба, Мохамед Салиу Ба, Джусуфу Пайе, Абдулай Конте, Сэм Саффа, Алие Тия, Мустафа Сонни, Эми Веиноглу, Джозеф Дж. Амон[3], Мэри Хамер Ходжес и Яо-Би Чжан

Реферат
Предпосылки: Базовое картирование кишечных червей (STH) передаваемых через почву среди детей школьного возраста (SAC) в 2008-2009 гг. показало их высокую или умеренную распространенность в 13 из 14 районов Сьерра-Леоне. Основываясь на этом исследовании, два раза в год на национальном уровне для детей дошкольного возраста (PSC) в возрасте 12–59 месяцев и периодически на субнациональном уровне для SAC проводилось массовое

введение лекарственного препарата (MDA) мебендазола/альбендазола. Кроме этого, MDA ивермектина и альбендазола для устранения лимфатического филяриатоза (ЛФ) проводятся по всей стране с 2010 года и предназначены для лиц старше пяти лет Массовое введение лекарственного препарата (MDA) проводилось широко за исключением 2014 года, когда все, кроме одного, раунды MDA для PSC были отменены из-за чрезвычайной ситуации, вызванной вирусом Эбола. Целью данного исследования было определение распространенности и интенсивности инфекции STH среди SAC после десятилетия кампаний дегельминтизации.

Методы: было целенаправленно выбрано семьдесят три школы в 14 районах , включая 39 школ из базового картирования, примерно с двумя участками из каждой категории (низкая, средняя и высокая) распространенности на базовом уровне в каждом районе. Образцы свежего стула были собраны у 3632 детей в возрасте 9-14 лет (мужского пола - 51%, женского пола - 49%) и исследованы с использованием метода Като Каца.

Результаты: Распространенность инфекций STH в Сьерра Леоне снизилась в 2016 году по сравнению с 2008: *Аскарис ламбрикод* 4.4% (95% доверительный интервал [*CI*]: 3.7–5.1%) против 6.6% (95% *CI*: 0–25%), *Тричирус тричиура* 0.7% (95% *CI*: 0.5–1.1%) против 1.8% (95% *CI*: 0–30.2%), нематода 14.9% (95% *CI*: 13.8–16.1) против 38.5% (95% *CI*: 5.4–95.1%), и любые STH 18.3% (95% *CI*:17.0–19.5%) против 48.3% (95% *CI*: 5.4–96.3%), соответственно. В 2016 году ни в одном округе не было высокой распространенности нематодов, а в четырех районах - она была умеренной, по сравнению с восемью и четырьмя районами, соответственно, в 2008 году. В 2016 году среднее арифметическое число яиц анкилостомы у всех обследованных детей было небольшим: 45,5 я/г (95% *CI*: 35,96–55,07 я/г); у трех (0,08%) детей были тяжелые инфекции, и у девяти (0,25%) детей были умеренные инфекции.

Выводы: Сьерра-Леоне добилась значительных успехов в борьбе с STH как проблемой общественного здравоохранения среди SAC. После прекращения LF MDA (между 2017 и 2021 гг.) необходимо ускорить переход дегельминтизации на другие платформы и стратегии в области водоснабжения и санитарии, чтобы сохранить контроль над STH и, в конечном итоге, остановить распространение инфекции.

Translated from English version into Russian by Natalia Bailey, Revised by Alexander Somin, through

TRANSLATORS WITHOUT BORDERS

La helmintiasis transmitida por el suelo en niños en edad escolar de Sierra Leona después de una década de intervenciones preventivas de quimioterapia

Yakuba Mohamed Bah, Mohamed Salieu Bah, Jusufu Paye, Abdulai Conteh, Sam Saffa, Alie Tia, Mustapha Sonnie, Amy Veinoglou, Joseph J. Amon[3], Mary Hamer Hodges y Yao-Bi Zhang

Resumen

Antecedentes: la determinación del nivel inicial de helmintiasis transmitidas por el suelo (HTS) en niños en edad escolar (NEE) en el período 2008-2009 señaló una incidencia alta o moderada en 13 de los 14 distritos de Sierra Leona. Después de estas investigaciones, se efectuó una administración masiva de los medicamentos (AMM) mebendazol/albendazol semestralmente a nivel nacional dirigida a niños en edad preescolar (NEP) de 12 a 59 meses y de manera intermitente a nivel subnacional dirigida a NEE. Asimismo, desde 2010 se ha llevado a cabo la AMM de ivermectina y albendazol a nivel nacional para eliminar la filariasis linfática (FL), dirigida a individuos de más de cinco años. Cada AMM alcanzó una alta cobertura, salvo en el año 2014, cuando fueron canceladas todas menos una ronda de AMM para NEP debido a la emergencia del Ébola. El objetivo de este estudio fue determinar la incidencia e intensidad de las infecciones por HTS en NEE después de una década de estas campañas de desparasitación.
Método: se seleccionaron deliberadamente 73 escuelas en 14 distritos, incluidas 39 escuelas de las encuestas de referencia, con aproximadamente dos puntos de cada categoría de incidencia (baja, media, alta) al inicio por distrito. Se recolectaron muestras de heces frescas de 3632 niños de 9 a 14 años (51 % de sexo masculino y 49 % de sexo femenino) y se examinaron mediante la técnica Kato Katz.
Resultados: la incidencia de HTS en Sierra Leona disminuyó en el año 2016 en comparación con 2008: *Ascaris lumbricoides* 4,4 % (intervalo de confianza [*IC*] del 95 %: 3,7–5,1 %) frente al 6,6 % (*IC* del 95 %: 0–25 %), *Trichuris trichiura* 0,7 % (*IC* del 95 %: 0,5–1,1 %) frente al 1,8 % (*IC* del 95 %: 0–30,2 %), anquilostomas 14,9 % (*IC* del 95 %: 13,8–16,1) frente al 38,5 % (*IC* del 95 %: 5,4–95,1 %), y cualquier HTS 18,3 % (IC *del 95 %*: 17,0–19,5 %) frente al 48,3 % (*IC* del 95 %: 5,4–96, 3%), respectivamente. En 2016, ninguno de los distritos presentó una alta incidencia de anquilostomas y cuatro distritos presentaron una incidencia moderada, en comparación con ocho y cuatro distritos respectivamente en el año 2008. En 2016, el promedio aritmético del conteo de huevos de anquilostomas en todos los niños examinados fue bajo: 45,5 hpg (*IC* del 95 %: 35,96–55,07 hpg); tres (0,08 %) niños presentaban infecciones fuertes y nueve (0,25 %) niños presentaban infecciones moderadas.
Conclusiones: Sierra Leona ha progresado notablemente hacia el control de las HTS como problema de salud pública en los NEE. A medida que se elimine gradualmente la AMM contra la FL (entre 2017 y 2021), la transición de las desparasitaciones a otras plataformas y estrategias de agua y saneamiento debe reforzarse para mantener el control de las HTS y, finalmente, interrumpir su transmisión.

Translated from English version into Spanish by Maria Gracia Zavarse, Revised by Mayra León, through

TRANSLATORS
WITHOUT BORDERS
